# Anti-HIV microRNA expression in a novel Indian cohort

**DOI:** 10.1038/srep28279

**Published:** 2016-06-20

**Authors:** Rakesh Dey, Kartik Soni, Shanmugam Saravanan, Pachamuthu Balakrishnan, Vikram Kumar, Jayaseelan Boobalan, Sunil Suhas Solomon, Vinod Scaria, Suniti Solomon, Samir K. Brahmachari, Beena Pillai

**Affiliations:** 1 CSIR-Institute of Genomics and Integrative Biology (CSIR-IGIB), Mathura Road, Delhi 110 020, India; 2 Academy of Scientific and Innovative Research (AcSIR), New Delhi, India; 3 Y R Gaitonde Centre for AIDS Research and Education (YRG CARE), VHS Campus, Rajiv Gandhi Road, Taramani, Chennai 600113, India; 4 Johns Hopkins University School of Medicine, Baltimore, USA

## Abstract

HIV-1 replication inside host cells is known to be regulated by various host factors. Host miRNAs, by virtue of its normal functioning, also regulate HIV-1 RNA expression by either directly targeting virus mRNAs or indirectly by regulating host proteins that HIV-1 uses for own replication. Therefore, it is highly possible that with differential miRNA expression, rate of disease progression will vary in HIV-1 infected individuals. In this study we have compared expression of a panel of 13 reported anti-HIV miRNAs in human PBMCs from long term non progressors (LTNPs), regular progressors and rapid progressors. We found that LTNPs have substantial lower expression of miR-382-5p that positively correlates with viral loads. Combinatorial regulation is highly probable in dictating differential disease progression as average expression of miR-382-5p and miR-155-5p can substantially distinguish LTNP individuals from regular progressors.

Human Immunodeficiency Virus-1 infects human cells and systematically evades the immune system while incapacitating it by causing the death of T-lymphocytes. The virus, after gaining entry through the intravenous route or lesions in mucosal surfaces binds to the CD4 receptor and the CCR5 or CXCR4 co-receptors on immune cells, penetrates the cell and releases its RNA genome which is reverse transcribed into DNA and integrated into the host genome. Viral particles then get disseminated from the infected founder population and spread out to new reservoirs of various tissue sites to establish systemic infection inside the host^1^. During the late stages of the infection, characterized by high viral load and low CD4+ T-cell counts in the blood, the patient becomes susceptible to infections by secondary pathogens like *Pneumocystis carinii*, *Candida albicans* and *Mycobacterium tuberculosis* and eventually succumbs to Acquired Immune Deficiency Syndrome (AIDS)^2^,^3^.

Host factors that reduce the cytotoxicity caused by the virus and viral factors required for effective infection are therefore prime targets for drug development. Genetic variations that confer resistance to the virus also hold the promise of cure through genome editing^4^,^5^,^6^. A small percentage (~3–4%) of HIV-1 infected individuals progress to AIDS relatively slowly^7^. Mutations in the viral genome that inactivate key genes required for replication result in slow progression as demonstrated in the case of the Sydney Blood Bank Cohort^8^. The hematopoietic stem cells from donors carrying a mutant CCR5 co-receptor in fact confer resistance in bone marrow transplant recipients^6^. These instances of HIV-1 suppression demonstrate that host factors can alter the course of HIV-1 infection and AIDS. An even rarer event could be the presence of yet unknown genetic variation that may confers resistance to HIV-1 in certain individuals leading to an “elite suppressor” status.

The first reports of anti-HIV-1 microRNAs, small regulatory RNAs that bind to the HIV-1 genome and prevent its expression and propagation, were made almost a decade back^9^,^10^. Several studies have since then shown the ability of these microRNAs to reduce virus replication in *in vitro* models of HIV-1 infection^11^,^12^,^13^,^14^. Although each reported anti-HIV miRNA has been validated by one or more research groups, there are no miRNAs universally acknowledged as anti-HIV miRNAs. These studies cannot be compared to each other easily because of difference in patient detection methods and candidate miRNAs studied. Thus there is a need for a comprehensive evaluation of anti-HIV miRNAs in a large patient cohort.

It is well known that eukaryotic mRNAs are often targeted by several miRNAs. Furthermore, miRNAs may show protective effects by both targeting HIV-1 genome and augmenting the host immune response or production of immune cells to replace cells lost due to viral cytotoxicity. Thus, it is important to study the expression of predicted and previously reported anti-HIV-1 miRNAs in parallel, to explore their combinatorial effects on viral load and T-cell numbers. However, it is technically not feasible to study the effect of miRNA combinations *in vitro* due to the large number of possible permutations and combinations. Patients with varied progression rates, with and without retroviral therapy provide a powerful alternative wherein the levels of several anti-HIV-1 miRNAs, T-cells and viral load monitored simultaneously can provide information about protective miRNA combinations. Further, such studies in large cohorts may reveal new genetic factors that confer protection from HIV-1, through the enhanced expression of anti-HIV-1 miRNAs. Comprehensive expression profiling of anti-HIV-1 miRNAs in several cohorts is required to identify such rare variants that could account for the variability in disease progression rates.

Here, we report the expression levels of 13 anti-HIV-1 miRNAs, in human PBMCs from a large Indian cohort of HIV-1 patients with a significant number of long-term non-progressors and couple of elite suppressors. Most of the Long Term Non-progressors do not carry known, protective, genetic factors implying that detailed studies in this cohort may reveal novel protective mutations. We find distinctive, large changes and increased variability in the expression of these miRNAs in infected individuals compared to uninfected individuals. A combination of expression profiles of two microRNAs showed a substantial difference between long term non-progressors and regular progressors. ART therapy showed strong changes in microRNA expression although not always restoring the expression pattern to that of uninfected controls.

## Materials and Methods

### Enrollment of patients and healthy volunteers

This is a cross sectional study including HIV-infected individuals (n = 72) and HIV negative controls (n = 19) were enrolled based on their eligibility at “YRG CARE”. Written informed consents were obtained from all the participants in the cohort. Absolute CD4 counts and viral load data were provided to the HIV positive participants. These participants (details in Table 1) were classified as (1) Long Term Non Progressors (LTNPs, n = 10), (2) Long Term Non Progressors-Non Controllers (LTNP.NC, n = 7), (3) Regular Progressors (n = 28) and (4) Rapid Progressors (n = 11) based on the CD4 counts and the number of years of progression after they were diagnosed as seropositive. Apart from these 4 types of conventional progressors, there are 16 patients in our cohort, whom we could not assign into any of the previously mentioned group, hence kept as “Ambiguous” class (n = 16), they need more duration of follow up to be assigned to any particular class). Experimental methods related to human subjects were approved by “Institutional review board” of “YRG CARE”. All molecular biology experiments were carried out in accordance with approved guidelines of “CSIR-IGIB”.

### Isolation of PBMC

We have collected 20 ml of peripheral venous blood in BD vacutainers (Heparin based) from HIV positive participants and 10 ml of peripheral blood from HIV negative controls. Following blood collection, peripheral blood mononuclear cells (PBMCs) were isolated through conventional density gradient centrifugation and stored for subsequent RNA and DNA isolation.

### DNA Isolation

Genomic DNA was isolated from PBMC samples of HIV-1 infected/uninfected healthy controls by QIAGEN^®^ DNeasy^®^ Blood & Tissue Kits, according to the manufacturer’s protocol. DNA concentration was determined by measuring absorbance at 260 and 280 nm using a spectrophotometer (Eppendorf, Hamburg, Germany). Quality of genomic DNA was also determined by agarose-gel electrophoresis.

### PCR Amplification and Sequencing

Selected regulatory genes or regions (e.g. CCR5Δ32 polymorphisms, HLA-B5701/2705, HIV-1 Nef) were PCR amplified for further analysis. For CCR5Δ32 polymorphisms PCR amplification was carried out by following primers 5′-TTTACCAGATCTCAAAAAGAAG-3′ (forward) and 5′-GGAGAAGGACAATGTTGTAGG-3′ (reverse) which gave a 274 bp product for wild type allele, and 242 bp product for CCR5Δ32 allele^15^. To amplify Nef from proviral genomic DNA, a nested PCR approach was considered^8^. Oligonucleotides for double nested PCR amplification were as following; for outer PCR, SK-68 (5′-AGCAGCAGGAAGCACITATGG-3′) and CI-6, (5′-TGCTAGAGATTTTCCACAC-3′) were used. A fraction of amplified PCR product was used as template for inner PCR; Nef (5′-GTAGCTGAGGGGACAGATAG-3′) and LTR-3 (5′-AGGCTCAGATCTGGTCTAAC-3′) which gave 862 bp amplified product. For HLA PCR, an established sequence specific primer typing (PCR-SSP) method was followed^16^. For HLA-Control, 5′-ATGATGTTGACCTTTCCAGGG-3′ (forward) and 5′-TTCTGTAACTTTTCATCAGTTGC-3′ (reverse) were used, to obtain 256 bp amplified product. To amplify HLA-B5701, oligonucleotides 5′-AACATGAAGGCCTCCGCG-3′ (forward) and 5′ CGTCGCAGCCATACATCAC 3′ (reverse) gave 351 bp amplified product. For HLA-B2705 PCR amplification, oligonucleotides 5′-GCTACGTGGACGACACGCT-3′ (forward) and 5′-TCTCGGTAAGTCTGTGCCTT-3′ (reverse) gave 150 bp amplified product.

### Isolation of Total RNA

Total RNA was isolated from PBMCs using TRIzol (Invitrogen, USA) according to the manufacturer’s protocol. The RNA pellets were washed with 70% ethanol and air dried. Pellets were re-suspended in Nuclease Free water (Ambion). RNA concentration and purity were determined by measuring absorbance at 260 and 280 nm using a spectrophotometer (Eppendorf, Hamburg, Germany) and through RNA specific dye in Qubit 2.0 based measurements.

### Profiling of miRNAs by Real Time PCR

25 ng of total RNA from each of the patient samples and uninfected volunteers (control samples) were used for cDNA preparation by QuantiMir based oligodT adapter. Real time PCR quantification of all 13 selected miRNAs was done by SYBR Green (Roche) chemistry and QuantiMiR Kit (System Biosciences) as per manufacturer’s protocol. We used three endogenous controls- U1, U6 and 5.8S RNA and normalized our Real Time PCR data with Geomean of all three endogenous controls.

### Analysis of miRNA profiling data

All the miRNA Ct values were normalized to geomean of endogenous controls’ (U1, U6 and 5.8S RNA) Ct values. ΔCt values of each of the miRNA for controls as well as for all the progressors were plotted as box plots (along with data points) using “Origin” (OriginLab, “ http://www.originlab.com/”) software. Student’s T tests (unpaired, two tailed) were performed between uninfected controls and patient classes or in between different patient classes to check statistical significance of the miRNA expression values (ΔCt).

### Principal Component Analysis (PCA)

ΔCt values of controls and patient classes were analyzed for identifying the presence of principal components that may segregate different classes of progressors as well as uninfected controls. There are 13 variables (ΔCt values of 13 miRNAs) in our data. PCA analysis were performed in Microsoft excel by add-in “Multibase” package (Numerical Dynamics, Japan).

## Results

### Genetic Characterization of the patients

**Figure 1:**
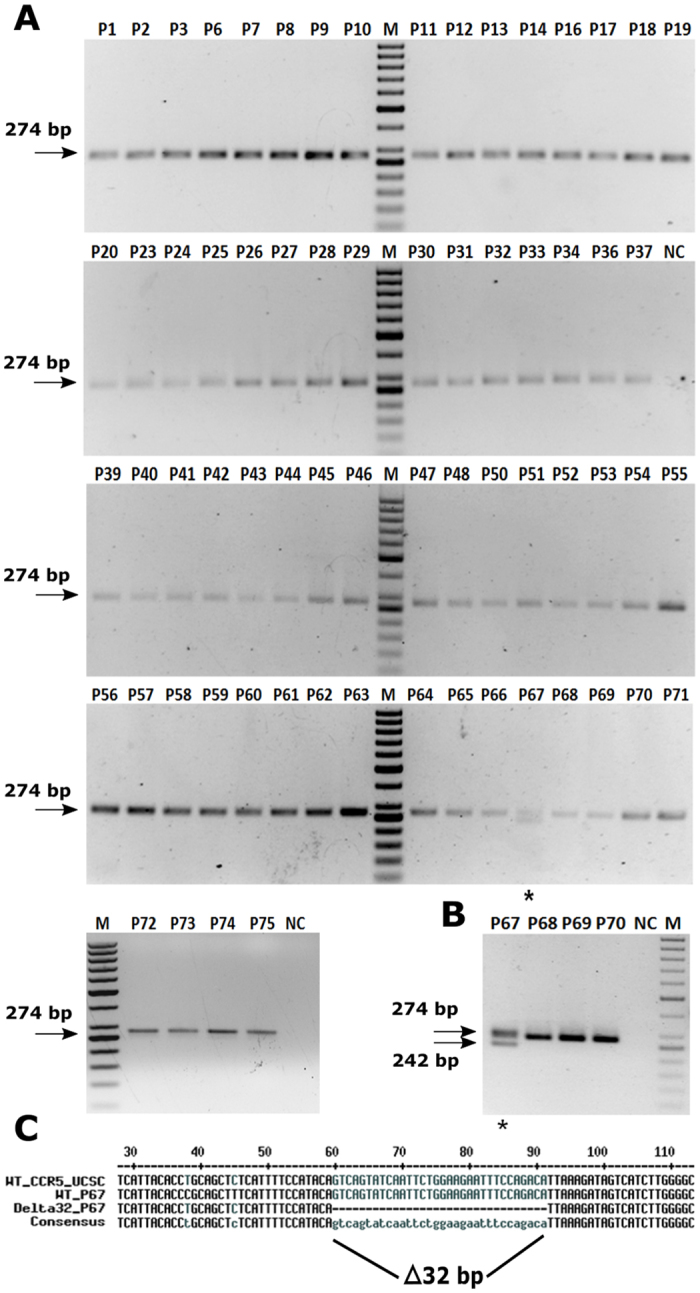
Characterization of CCR5Δ32 mutation status in the patient cohort; patient numbers are indicated above the respective lanes; NC = no-template PCR control; M = marker. Genomic DNA was isolated from the PBMCs of 67 patients and subjected to PCR using primers flanking the CCR5Δ32 mutation such that the normal allele would produce a 274 bp product while the mutant would give rise to a 242 bp product (**A**). The only heterozygote patient shows both bands; see technical replicate (**B**) for clear bands. The PCR products from patient P67 were separated, purified and sequenced to confirm the presence of the CCR5Δ32 mutation (**C**).

**Figure 2:**
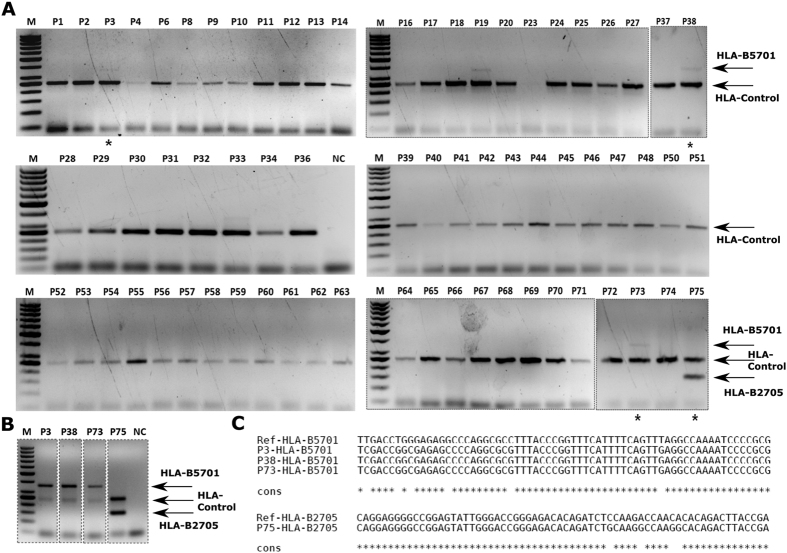
Characterization of HLA types in the patient cohort; patient numbers are indicated above the respective lanes; NC = no-template PCR control; M = marker. Genomic DNA was isolated from the PBMCs of 67 patients and subjected to PCR using primers flanking the respective regions, such that the common allele, HLA Control would produce a distinct band at 256 bp while HLA-B5701 would produce a 351 bp band and HLA-B2705 would produce a 150 bp band. Patient P75 showed the presence of a HLA-B2705 allele and patient P3, P38 and P73 showed HLA-B5701 alleles, see technical replicate (**B**) for clear bands, which were further confirmed by sequencing (**C**).

Differential rate of HIV disease progression in different classes of progressors, are known to be associated with various host and viral factors^17^. Among those, the most common three factors that have been repeatedly associated with rate of disease progression are (i) genetic (CCR5Δ32 mutation), (ii) immunologic (HLA-B5701/B2705) and (iii) viral (nef deletion/mutation). We sought to study the contribution of any of these factors to the progression of disease in the patients of the current cohort (Table 1). We have isolated genomic DNA from PBMCs of all the seropositive as well as seronegative individuals. We performed PCR amplification of specific HLA alleles (HLA Control, HLA-B5701 and HLA-B2705), CCR5 and Nef regions to find out presence of any such regulatory factors for slower disease progression. From PCR amplification and agarose gel electrophoresis, we did not find any patient positive for CCR5Δ32 homozygous allele (Fig. 1). One patient (1/67, 1.49%) with regular progression of disease possesses CCR5Δ32 heterozygous allele, which matches with the expected population frequency of CCR5/CCR5Δ32 individuals in India (1.47–4.69%)^18^. Three patients are positive for HLA-B5701 alleles (3/68, 4.41%) and one patient positive for HLA-B2705 allele (1/68, 1.47%), in synchrony with expected allele frequencies in Indian population^19^ (Fig. 2). PCR amplification and gel electrophoresis of the HIV-1 Nef region show that all 43 individuals (among the 43 patient screened) contain wild type Nef gene copies (Supplementary Figure 1).

### Anti-HIV miRNAs

**Figure 3:**
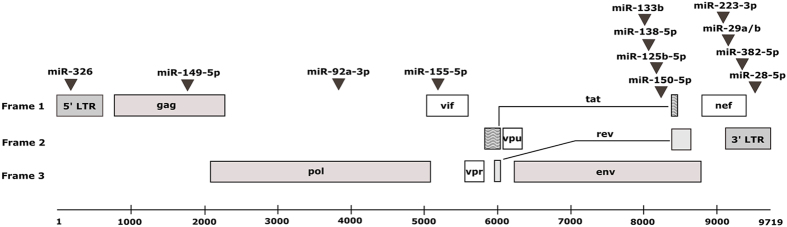
Shortlisted 13 anti-HIV miRNAs and their reported target regions on HIV-1 genome.

Jeang *et al*. compiled a list of reported anti-HIV miRNAs with direct and indirect roles^20^. We used this as a starting point to shortlist 13 such anti-HIV miRNAs that have direct target site/s on HIV-1 genome (Fig. 3). Other groups have previously used microarrays or qRT-PCR based accurate but expensive assays like Taqman for detection of single miRNAs across many samples or miRnome arrays for profiling of limited samples. Given the size of our cohort and our objective of monitoring all the reported anti-HIV miRNAs in parallel, we preferred to use a highly specific but multiplex assay based on tailing the endogenous miRNAs and detecting the resulting long RNA products using a universal reverse primer and miRNA specific forward primers (see methods for details). We also found that miRNAs like miR-92-3p that are considered house-keeping miRNAs and have been used previously for normalization are not suitable for this study because of their reported involvement in AIDS. We used geometric mean of the expression level of U1, U6 and 5.8 s, after confirming that these RNAs showed minimal variation between samples (Table 2). We found that starting with 25 ng of total RNA isolated from PBMCs, we consistently detected all the miRNAs in a Ct value range of 17–33, in uninfected controls. The miRNAs were also consistently detectable in patient samples, but at higher Ct values. We subsequently used ΔCt values for all further analysis.

### HIV infection causes differential expression of host miRNAs

**Figure 4:**
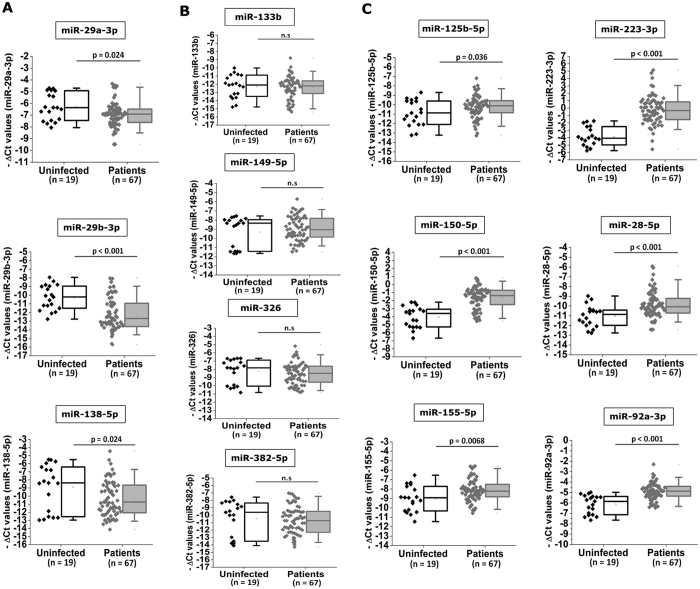
(**A**) Three miRNAs (miR-138-5p, miR-29a-3p, and miR-29b-3p) were down regulated, (**B**) four miRNAs (miR-133b, miR-149-5p, miR-326, and miR-382-5p) remain mostly unchanged, and six miRNAs (miR-125b-5p, miR-150-5p, miR-155-5p, miR-28-5p, miR-223-3p, and miR-92a-3p) were up regulated in patients, compared to uninfected controls. P values were calculated through unpaired, two tailed T tests.

We found that anti-HIV miRNAs are differentially expressed in HIV-1 infected cohort compared to non- infected healthy individuals (Fig. 4; Table 3). Amongst the 13 miRNAs we selected, 6 miRNAs (miR-28-5p, miR-125b-5p, miR-150-5p, miR-155-5p, miR-223-3p, and miR-92a-3p) show up-regulated expression and 3 miRNAs (miR-29a-3p, miR-29b-3p, and miR-133b) show down-regulation of expression in patient samples, compared to uninfected counterparts. Four miRNAs (miR-138-5p, miR-149-5p, miR-326, and miR-382-5p) show non-significant changes. These miRNAs were not merely an indirect indicator of T-cell status, because they showed no significant difference in patients with higher T-cell counts compared to patients with low T-cell counts (data not shown).

### miRNA expression changes amongst different progressors

**Figure 5:**
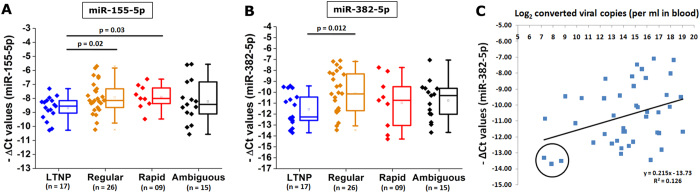
(**A**) Expression values of miR-155-5p and (**B**) miR-382-5p were plotted among four classes of progressors. MicroRNA-155-5p showed lower expression in LTNPs compared to Regular/Rapid progressors (**A**). For miR-382-5p, LTNPs showed lower expression values compared to Regular progressors (**B**). Expression of miR-382-5p among LTNPs, LTNP_NC and regular progressors (both Naïve and ART positive) showed a positive correlation with viral load values (**C**). Together, n = 43 and three LTNP individuals (P6, P12 and P30, encircled in black) having among the lower most miR-382-5p values also have close to undetectable viral loads.

Our cohort includes patients with distinct differences in rate of progression of disease. Of the patients, 17 HIV-1 positive individuals (6 males, 11 females, all drug naïve) have CD4+ T cell counts >=500 for >=7 years duration at the time of sampling. The cohort also consisted of 28 patients (13 males, 15 females, drug naïve and ART positive together) who showed regular rate of progression and 11 patients (4 males, 7 females) who showed rapid progression, with CD4+ T-cells plummeting below 300 within first 3–5 years. When we compared the expression of the anti-HIV miRNAs among four patients classes (segregated into LTNPs, Regular, Rapid and Ambiguous), we found (Fig. 5A,B) miR-155-5p and miR-382-5p showed a significant (p < 0.05) down regulation in LTNPs when compared to Rapid/regular progressors. The fold repression, (at 2.5 fold) was highest when miR-382-5p expression is compared between LTNPs and Regular Progressors while miR-155-5p showed a 1.8 fold down regulation in LTNPs when separately compared to both regular and rapid progressors. We also looked for the status of the elite suppressors to check if they were outliers in expression of any of the anti-HIV miRNAs. One of the elite suppressors showed very low expression of miR-382-5p, but barring this case, no general conclusion could be drawn for the elite suppressors. Lastly, we looked for an overall correlation between the expression level of each miRNA and the viral load in patients. We found that expression of miR-382-5p showed positive correlation with viral load amongst LTNPs (Fig. 5C). Three LTNP samples (P06, P12 and P30, encircled in black) with very low viral loads (<150, 232 and 413 copies/ml) showed a strong repression of miR-382-5p.

**Figure 6:**
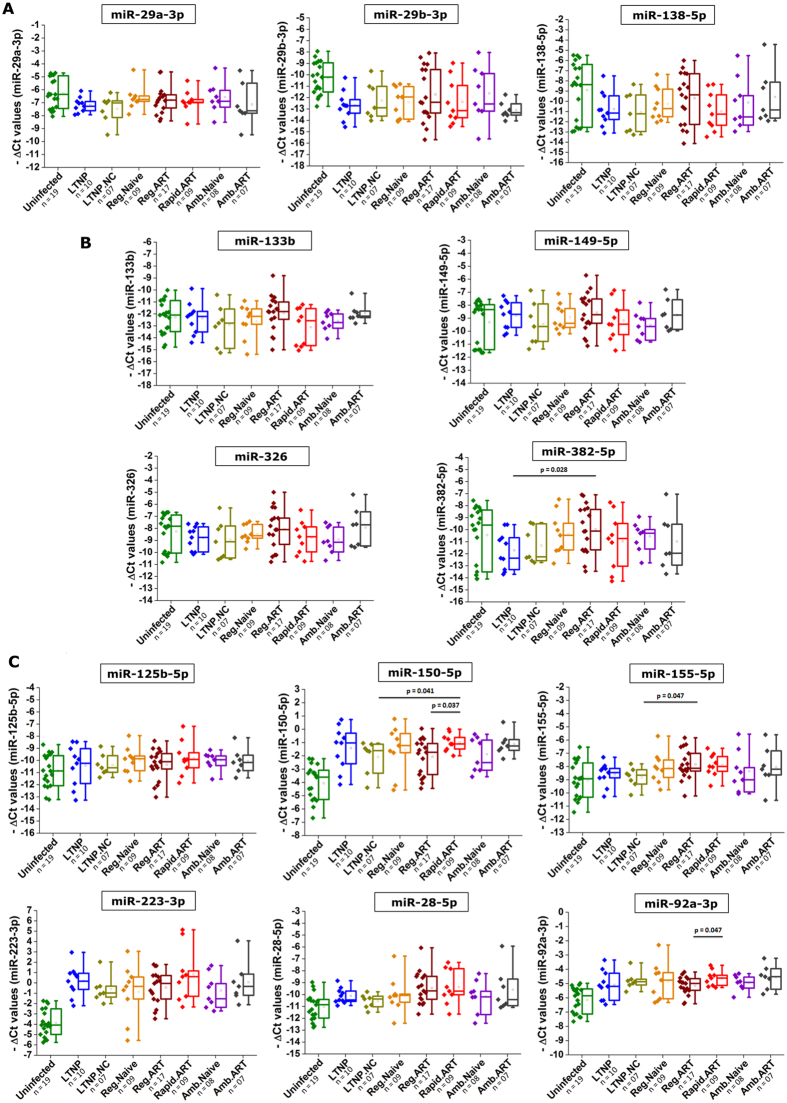
Expression values of all 13 miRNAs are plotted among all classes (segregated and clustered into sub-classes based on viral loads and ART status). (**A**) Down regulated miRNAs (as Fig. 4A), (**B**) unchanged miRNAs (as Fig. 4B), and (**C**) Up regulated miRNAs (as Fig. 4C). Only significant expression differences among various classes are marked with p values on the graphs. P values were calculated as unpaired two tailed T tests and p values < 0.05 were considered significance.

Apart from having a steady CD4+ T cell counts (>=500) for >=7 years duration, strong LTNPs also represses the viral replication below 10,000 copies/ml of blood. Among the 17 LTNP individuals, 10 patients successfully kept the viremia <10,000 copies/ml blood. The remaining 7 LTNP patients have >10,000 viremia counts (sub-grouped as LTNP_NC). When we segregated our patients into drug naïve and treated subgroups and compared the miRNA expression patterns, we found, the expression profile of any single miRNA is not statistically significant to distinguish different progressor classes (Fig. 6). However, four miRNAs (miR-382-5p, miR-155-5p, miR-150-5p and miR-92a-3p) showed differences in a few patient subgroups. LTNP individuals have down-regulated expression (Fig. 6A) of miR-382-5p compared to regular progressors (ART positive). LTNP_NC patients vary significantly (Fig. 6B) in miR-155-5p expression compared to Rapid progressors. Rapid progressors (ART positive) have higher expression of miR-150-5p (Fig. 6C) compared to both regular progressors (ART positive) and LTNP_NC (drug naive). Regular progressors (ART positive) have lower expression (Fig. 6C) of miR-92a-3p compared to rapid progressors (ART positive).

### Combinatorial expression of selected miRNAs correlates with rates of disease progression

**Figure 7:**
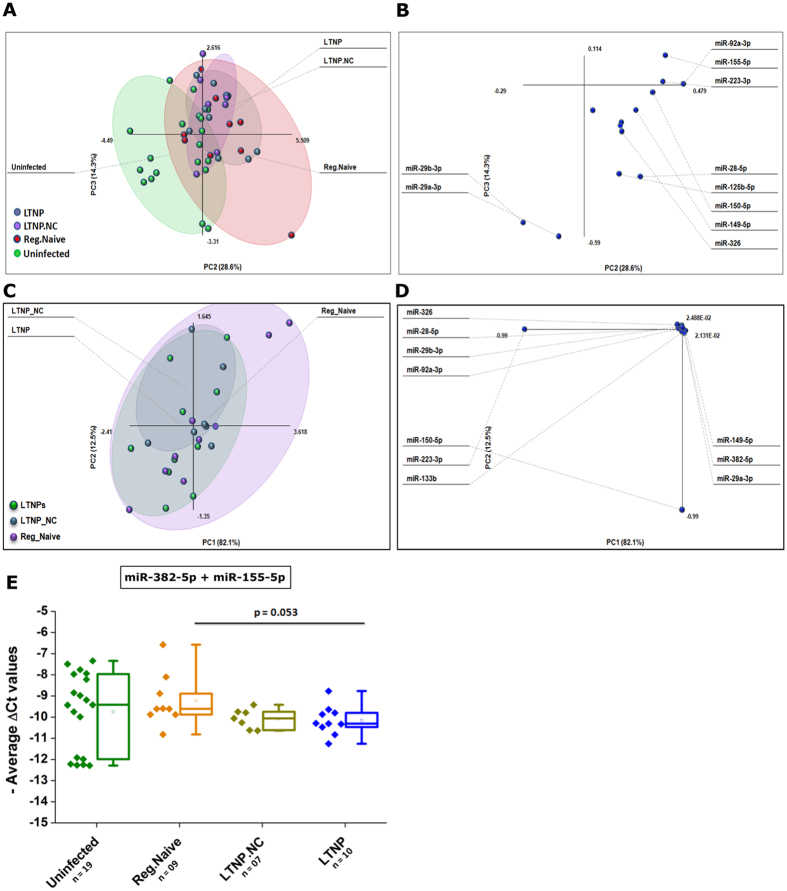
(**A**) Principle component analysis was done in Microsoft Excel by add-in “Multibase” package, using all 13 miRNA expression values (ΔCt) as variables. LTNPs and LTNP_NCs are clearly segregated from uninfected controls (**A**), while Regular_Naive individuals overlap partially on the 2D PCA plot (PC2 vs PC3). (**B**) The overall variance and 10 mostly contributing miRNAs in these two components. (**C**) PCA was done among 3 disease progressors classes. (**D**) The overall variance and 10 mostly contributing miRNAs in these two components. (**E**) LTNPs show substantially lower expression from Regular progressors, when average “−ΔCt” values of two miRNAs (miR-382-5p and miR-155-5p) were plotted.

Next, we investigated whether we can identify any signature expression pattern with any combination (from the panel of 13 miRNA) of miRNAs that show correlation with disease progression. Principal component analysis with 13 variables (ΔCt values of 13 miRNA, Fig. 7A) among uninfected controls and three drug naïve patient classes (LTNPs, LTNP-NCs and Regular_Naive) revealed the contribution and relation among these 10 miRNAs (Fig. 7B). A 2D plot (PC2 vs PC3) showed that uninfected individuals (red dots) are separated well from the LTNPs and LTNP_NCs. Regular_Naive individuals falls between LTNPs and uninfected individuals, and there is overlap between LTNP_NC and LTNP patients. PCA among three patient classes (LTNPs, LTNP_NC and Regular_Naive) do not show a good separation among patient classes (Fig. 7C,D) in a 2D plot (PC1 vs PC2), however these PCAs gave us clues about contributing miRNAs, responsible for maximum variances in different components. Next, we investigated, whether we can segregate the patient classes based on average expression pattern of miRNAs. Empirically, we found that average expression of two miRNAs, miR-382-5p and miR-155-5p can substantially distinguish LTNP individuals from regular progressors (Fig. 7E).

## Discussion

Since its discovery, Human Immunodeficiency Virus (HIV) manages to snatch away one of the highest number (https://www.aids.gov/hiv-aids-basics/hiv-aids-101/global-statistics/) of human lives, caused by infectious diseases. After three decades of research, although we are now capable of controlling the severity of disease progression and improving the lifespan of HIV infected individuals, there is no practical cure. The strategy HIV adopted is to infect and kill the host’s CD4+ T cells and antigen presenting cells, so as to dismantle the innate-adaptive immune balance and make the host susceptible to secondary opportunistic co-infections. To control this threat, host also has its own arsenal of restrictive factors that creates a tug-of-war to gain control on each other^21^. These initial events of host-virus interactions establish the balance and thus regulate the rate of disease progression later in the course of infection. Current approach to deal with HIV infection is the use of combinatorial anti retroviral drug therapy (ART) that targets more than one stages of HIV life cycle. Besides being a successful approach in terms of reducing the viral load and improving the life span of infected individuals, ART poses a few important concerns that call for development of alternative approaches to treat HIV infection^22^. In quest of search of important host/viral factors, a significant number of reports have pointed out various proteins, enzymes and non-coding RNAs that play important roles in different stages of HIV life cycle^12^,^21^,^23^.

MicroRNA, a subset of 19–24 nucleotides of non coding RNA has been a major player in regulation of gene expression during development and disease^24^. By virtue of miRNAs working mechanisms, our group previously hypothesized that host miRNA might be able to target the HIV RNA genome. Subsequent analyses through bioinformatics prediction pointed out the presence of host miRNA target sites on HIV-1 genome. This initial finding led us to explore the effect of real interactions among the host miRNA-HIV-1 targets that might alter the normal progression of the disease. Later, a study by our group also validated the interactions of miR-29a/b and HIV-1 *Nef*, an accessory protein of the virus, required for viral replication inside host cells^25^,^26^. An interesting finding by Huang *et al*. suggested that a cluster of miRNAs (miR-28-5p, miR-125b-5p, miR-150-5p, miR-223-3p and miR-382-5p) have enriched expression in resting primary CD4+ T lymphocytes and have direct target sites on HIV-1 mRNA, thus leading to differential HIV-1 infectivity/resistance to different cells lying at varying developmental stages^11^. Since then, a few more host miRNAs with direct target sites on HIV-1 genome have been reported^20^. Different groups have looked at the miRNA expression profile and tried to predict if any correlation to disease progression could be drawn. Throughout these studies, cellular miRNAs were found to be differentially expressed among different classes of seropositive individuals who show differential disease progression following HIV-1 infection^27^,^28^,^29^,^30^,^31^,^32^. However, our understanding of miRNA expression pattern and any possible correlation with disease progression is still rudimentary.

In this study, we have measured a pool of host miRNA expression levels in PBMCs of a cohort of HIV-1 infected South Indian individuals and drawn a possible correlation with differential disease progression patterns in different categories of patients. Based on the previous cumulative evidence and a recent useful review by Klase *et al*.^20^, we choose a panel of 13 miRNAs, which have direct target sites on HIV-1 genome and are shown to be important regulators of HIV replication in *in vitro* studies. Since our study is restricted to PBMCs, we could not monitor the levels of circulating miRNAs in the plasma which has been recently reported to be important^33^. Similarly, the possibility that altered differentiation patterns or factors like immune activation levels, cellular exhaustion, and senescence affect the total PBMC expression levels cannot be ruled out. In spite of this caveat, total PBMCs are useful in identifying outliers within patient groups with excessively altered expression level of specific miRNAs. Since HIV infected PBMCs are a small population of the total PBMCs, the baseline expression of anti-HIV miRNAs in outliers may reflect genetic factors that confer resistance. In a retrospective study we cannot rule out the possibility that time dependent changes in the cell counts maybe correlated to the detected miRNA levels. In future, longitudinal studies may be useful in tracking time-dependent changes in immune cell sub-populations.

For *in vivo* replication, HIV is solely dependent on the host cellular machinery. To create a favorable environment to survive and replicate, HIV modulates the expression of many host proteins. On the other hand, host also regulates HIV by modulating several of these factors. Such regulation can be either by down regulating factors that HIV uses in its favor or by up regulating restriction factors in order to create a hostile environment for HIV. MicroRNAs, the modulators of gene expression are part of this intricate host-virus relationship and can be utilized in order to shift the balance. The readout of miRNA expression among different types of disease progressors has an advantage as it might show correlation with a particular situation and can be interpreted as either favorable to virus or host. This has potential therapeutic values, as by tuning the expression of miRNAs, a favorable situation for the host can be gained. For understanding the anti-HIV role of miRNAs, it is important to classify the infected individuals as homogenously as possible. In our study, while comparing miRNA expression, we have clustered the patients on the basis of viral loads (LTNPs and LTNP_NC) and also by ART status (among regular progressors). This is important to negate out factors that might result in high variability in miRNA expression. We have also characterized our patient cohort in order to find any known factors (CCR5Δ32, HLA-B5701/2705 and HIV-1 Nef) that associate with slower disease progression. There are only 4 patients (three are HLA-B5701 positive and one is HLA-B2705 positive) who have protective HLA alleles. Therefore, our study cohort is mostly independent of host/viral factors that affect disease progression.

HIV infection has shown to alter expression of many host miRNAs^27^, which is a complex outcome of many direct or indirect interactions taking place inside the infected cell. In our study, there are 6 miRNAs (miR-28-5p, miR-125b-5p, miR-150-5p, miR-155-5p, miR-223-3p and miR-92a-3p) that are up regulated in patients (compared to uninfected controls). MicroRNA-223-3p was also shown to be up regulated in HIV infection in an *in vitro* study, previously^34^. Up regulation of these miRNAs were shown to be anti-correlated with susceptibility of HIV infection in monocytes/macrophages^35^. The results from our study (in the cohort) are in synchrony with previously reported *in vitro* findings. We speculate that this up regulation of anti-HIV miRNAs is a manifestation of host control mechanisms in order to repress the viral replication. Also, 3 miRNAs (miR-138-5p, miR-29a-3p, and miR-29b-3p) are down regulated in patient samples. Down regulation of anti-HIV miRNAs can be in favor of the host, if those miRNAs regulate HIV in an indirect fashion. MicroRNA-382-5p is shown to be enriched in primary CD4+ T lymphocytes, where it contributes to HIV-1 latency^11^. We propose that individuals with lower miR-382-5p are better at clearing the virus infected reservoir, thus in turn repressing the viral replication. As this was not a longitudinal study, we cannot comment on the temporal changes in expression of miRNA during infection or their role in latency.

In summary, we report here an evaluation of previously reported anti-HIV miRNAs in a novel Indian cohort comprising HIV infected individuals with different rates of disease progression. Our results indicate that reduced expression of miR-382-5p and miR-155-5p may be critical in establishing slow progression.

## Additional Information

**How to cite this article**: Dey, R. *et al*. Anti-HIV microRNA expression in a novel Indian cohort. *Sci. Rep.* **6**, 28279; doi: 10.1038/srep28279 (2016).

## Supplementary Material

Supplementary Information

## Figures and Tables

**Table 1:** Classification criteria and details of the cohort.

	**Long Term Non Progressors (LTNPs)**	**Long Term Non Progressors_non controllers (LTNP.NC)**	**Regular Progressors**	**Rapid Progressors**	**Ambiguous**	**Un-infected controls**
Number of Individuals	10	7	28	11	16	19
CD4 Counts	≥500	≥500	500 > CD4 Counts ≥ 300	<300	wide range, do not fall into rest 4 classes	–
Progression (in years)	≥7 years	≥7 years	≤5–7≤	<5 years	wide range, do not fall into rest 4 classes	Not Applicable
Viral Load (median values)	1164	22520	124627, 40302	229543	12349, 40640	Not Applicable
Treatment status	All Naive	All Naive	Naïve = 09 On ART = 19	All on ART	Naïve = 09 On ART = 07	Not Applicable
Median Age(Range)	36 (21–47)^#^	24 (20–45)^#^	30 (22–49)^#^	31 (20–47)^#^	29 (17–45)^#^	27 (20–47)
Sex^##^	Female = 07 Male = 03	Female = 04 Male = 03	Female = 15 Male = 13	Female = 07 Male = 04	Female = 11 Male = 05	Female = 09 Male = 10
CCR5 status	CCR5 WT (n = 10)	CCR5 WT (n = 7)	CCR5 WT (n = 26) CCR5Δ32 heterozygous (n = 1)	CCR5 WT (n = 12)	CCR5 WT (n = 17)	Not tested
HLA-B5701/B2705 status	HLA-B5701 (n = 1) HLA-B2705 (n = 1)	None	HLA-B5701 (n = 1)	None	HLA-B5701 (n = 1)	Not tested

^#^p values (derived from t-test with uninfected controls) > 0.05.

^##^p values (derived from Chi-squared test with uninfected controls) > 0.05.

**Table 2:** Normalization controls and respective Ct values used in the miRNA expression profiling.

	Uninfected Control (n = 19)*	HIV positive patient (n = 67)*
**U1**	20.50–11.70 (16.88 ± 2.85)	24.56–16.74 (20.12 ± 1.99)
**U6**	23.89–16.75 (21.66 ± 1.93)	29.74–21.21 (24.02 ± 1.87)
**5.8 S**	17.93–10.30 (15.02 ± 2.40)	23.23–15.51 (18.35 ± 1.61)
*Range (mean ± SD)		

**Table 3:** Anti-HIV-1 miRNAs and respective Ct values in controls and patients.

	Uninfected Control (n = 19)*	HIV positive (n = 67)*	**miRNA sequence**
miR-28-5p^11^	32.88–24.36 (28.52 ± 2.68)	33.87–27.59 (30.51 ± 1.60)	AAGGAGCUCACAGUCUAUUGAG
miR-29a-3p^12^	27.13–19.22 (23.77 ± 3.00)	31.82–24.55 (27.61 ± 1.75)	UAGCACCAUCUGAAAUCGGUUA
miR-29b-3p^12^	31.42–21.92 (27.78 ± 3.20)	37.74–28.37 (32.84 ± 2.19)	UAGCACCAUUUGAAAUCAGUGUU
miR-92a-3p^14^	26.41–20.29 (23.63 ± 1.58)	29.70–22.58 (25.59 ± 1.85)	UAUUGCACUUGUCCCGGCCUGU
miR-125b-5p^11^	31.61–26.06 (29.68 ± 1.51)	35.25–27.04 (30.81 ± 1.89)	UCCCUGAGACCCUAACUUGUGA
miR-133b^14^	32.39–23.20 (28.44 ± 3.18)	39.29–28.87 (33.03 ± 1.77)	UUUGGUCCCCUUCAACCAGCUA
miR-138-5p^14^	28.42–24.08 (26.39 ± 1.52)	34.15–26.02 (30.97 ± 2.01)	AGCUGGUGUUGUGAAUCAGGCCG
miR-149-5p^14^	29.77–24.57 (26.80 + 1.09)	33.93–26.46 (29.64 ± 1.34)	UCUGGCUCCGUGUCUUCACUCCC
miR-150-5p^11^	25.28–17.84 (21.55 + 2.77)	26.78–18.65 (22.32 ± 2.01)	UCUCCCAACCCUUGUACCAGUG
miR-155-5p^36^	29.22–24.37 (26.53 + 1.56)	33.39–25.90 (28.86 ± 1.68)	UUAAUGCUAAUCGUGAUAGGGGU
miR-223-3p^11^	24.92–17.45 (21.37 + 2.51)	26.71–16.92 (20.92 ± 2.17)	UGUCAGUUUGUCAAAUACCCCA
miR-326^14^	28.06–23.75 (25.75 + 1.13)	32.39–25.17 (29.19 ± 1.47)	CCUCUGGGCCCUUCCUCCAG
miR-382-5p^11^	29.68–25.90 (27.94 + 1.19)	34.99–27.12 (31.42 ± 1.90)	GAAGUUGUUCGUGGUGGAUUCG
*Range (mean + SD)			
