# Supplementary material for: Anti-HIV microRNA expression in a novel Indian cohort
Source: Sci Rep. 2016 Jun 20;6:28279. doi: 10.1038/srep28279 (PMC4913240; doi:10.1038/srep28279)
Supplement: Supplementary Information [file srep28279-s1.pdf]

Supplementary Information

Title:

Anti-HIV microRNA expression in a novel Indian cohort

Authors:

Rakesh Dey[1,4], Kartik Soni[1], Shanmugam Saravanan[2], Pachamuthu Balakrishnan [2], Vikram Kumar[1], Jayaseelan Boobalan[2], Sunil Suhas Solomon[2,3], Vinod Scaria[1,4], Suniti Solomon[2], Samir K Brahmachari[1,4] & Beena Pillai[1,4]

Affiliations:

1. CSIR-Institute of Genomics and Integrative Biology, Mathura Road, Delhi 110 020, India.

2. Y R Gaitonde Centre for AIDS Research and Education, VHS Campus, Rajiv Gandhi Road, Taramani, Chennai 600113, India.

3. Johns Hopkins University School of Medicine, Baltimore, USA.

4. Academy of Scientific and Innovative Research (AcSIR), New Delhi, India.

Corresponding Author: Beena Pillai (beena@igib.in)

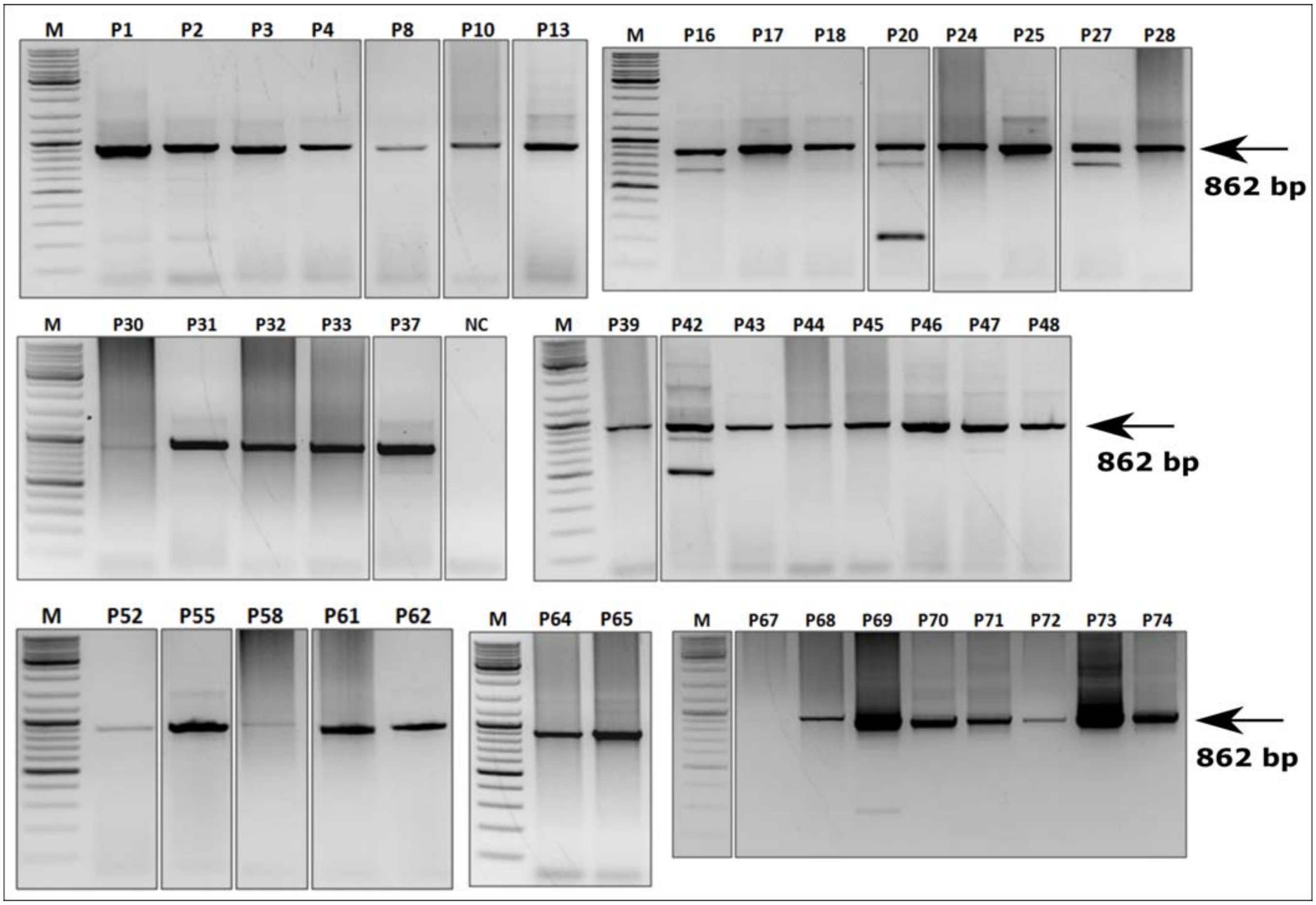

Supplementary figure 1 legend:

Characterization of HIV-1 nef mutation status in the patient cohort; patient numbers are indicated above the respective lanes; NC=no-template PCR control; M=marker. Genomic DNA was isolated from the PBMCs of 43 patients and subjected to nested PCR using primers flanking the nef region such that the wild type nef would produce a 862bp product.

Supplementary table 1: Regression Analysis - miR-382-5p

SUMMARY OUTPUT

Regression Statistics	
Multiple R	0.415201
R Square	0.172392
Adjusted R Square	0.058239
Standard Error	1.847633
Observations	67

ANOVA

	df	*SS*	*MS*	*F*	*Significance F*
Regression	8	41.24306	5.155383	1.510183	0.173666924
Residual	58	197.9973	3.413747		
Total	66	239.2404			

	Coefficients	*Standard Error*	*t Stat*	*P-value*	*Lower 95%*	*Upper 95%*	*Lower 95.0%*	*Upper 95.0%*
Intercept	-11.6048	2.645506	-4.38663	4.92E-05	-16.90040057	-6.30929	-16.9004	-6.30929
Match	0.01998	0.010955	1.823786	0.073338	-0.001949321	0.04191	-0.00195	0.04191
Hb	0.134347	0.100699	1.334148	0.18737	-0.067223356	0.335917	-0.06722	0.335917
Platelet	0.001419	0.003628	0.391178	0.697099	-0.005843612	0.008682	-0.00584	0.008682
WBC	0.141227	0.156418	0.902881	0.370324	-0.171878157	0.454332	-0.17188	0.454332
TLC	-0.38224	0.494607	-0.77282	0.442766	-1.372306986	0.60782	-1.37231	0.60782
CD4	-0.00203	0.003018	-0.67366	0.503204	-0.008074997	0.004008	-0.00807	0.004008
CD4%	-0.00507	0.086541	-0.05857	0.953498	-0.178299799	0.168163	-0.1783	0.168163
CD8_impute	-0.00073	0.00092	-0.78994	0.432782	-0.002567915	0.001115	-0.00257	0.001115

RESIDUAL OUTPUT

Observation	*Predicted miR-382-5p*	*Residuals*
1	-10.8898	-2.79495
2	-11.0195	1.353612
3	-10.4734	-1.54073
4	-11.3843	1.833175
5	-11.0136	-1.93609
6	-10.0543	3.007823
7	-10.1791	-1.78123
8	-11.0538	-0.5546
9	-10.2766	-0.7969
10	-9.95216	-0.34641

11	-10.8139	0.827673
12	-11.5806	1.385701
13	-10.6831	-2.07604
14	-10.0293	1.106242
15	-10.0688	0.425826
16	-12.0065	-1.70033
17	-11.2383	1.652194
18	-12.4239	-0.89981
19	-9.39931	-1.27559
20	-11.7074	-1.81194
21	-10.8703	-1.55801
22	-12.4669	0.098209
23	-9.8977	0.315056
24	-11.7849	0.912146
25	-10.0946	-1.05484
26	-10.5374	1.120504
27	-10.7481	0.310168
28	-11.0255	-1.55521
29	-10.4708	-1.78854
30	-11.6476	-1.07743
31	-12.384	0.067703
32	-11.544	2.036215
33	-9.85634	-0.58182
34	-10.8949	-0.17674
35	-10.8756	-3.06361
36	-12.3089	-1.97564
37	-10.3421	2.271243
38	-10.4097	-2.63438
39	-11.0213	0.283975
40	-10.2852	2.558925
41	-9.99562	0.503955
42	-10.8031	0.679517
43	-10.344	2.025833
44	-11.9352	4.750568
45	-10.5771	2.824429
46	-9.83245	2.732471
47	-10.4033	-2.67604
48	-11.0671	-0.60242
49	-10.8408	-0.93053
50	-11.3432	-2.11277
51	-10.2365	-1.6007
52	-10.0556	1.843777
53	-10.9143	2.549254
54	-9.9965	-0.15324
55	-10.5535	-1.12555
56	-10.7356	-0.01399
57	-9.52063	0.697767

58	-9.41344	0.029097
59	-11.8046	4.333868
60	-10.4391	-1.24784
61	-10.0224	-0.02088
62	-9.28738	1.257223
63	-10.5187	1.051936
64	-10.2535	-1.48313
65	-9.98757	-0.48477
66	-10.3065	-1.15095
67	-12.5524	-0.26243

y = B17+B18*X1+B19*X2+B20*X3 etc

Supplementary table 2: Regression Analysis - miR-155-5p

SUMMARY OUTPUT

Regression Statistics	
Multiple R	0.503036
R Square	0.253045
Adjusted R Square	0.150017
Standard Error	1.062275
Observations	67

ANOVA

	df	*SS*	*MS*	*F*	*Significance F*
Regression	8	22.17204	2.771505	2.456074	0.023071
Residual	58	65.44886	1.128429		
Total	66	87.6209			

	Coefficients	*Standard Error*	*t Stat*	*P-value*	*Lower 95%*	*Upper 95%*	*Lower 95.0%*	*Upper 95.0%*
Intercept	-3.42351	1.521003	-2.25083	0.028202	-6.46813	-0.37889	-6.46813	-0.37889
Match	-0.00108	0.006299	-0.17086	0.864932	-0.01368	0.011532	-0.01368	0.011532
Hb	-0.04346	0.057896	-0.75062	0.455919	-0.15935	0.072433	-0.15935	0.072433
Platelet	-0.00146	0.002086	-0.69854	0.487629	-0.00563	0.002719	-0.00563	0.002719
WBC	-0.066	0.089931	-0.73392	0.465954	-0.24602	0.114014	-0.24602	0.114014
TLC	-0.13376	0.284369	-0.47037	0.639853	-0.70298	0.435466	-0.70298	0.435466
CD4	0.003111	0.001735	1.792644	0.078245	-0.00036	0.006584	-0.00036	0.006584
CD4%	-0.14993	0.049756	-3.01325	0.003828	-0.24952	-0.05033	-0.24952	-0.05033
CD8_impute	-0.00105	0.000529	-1.99265	0.051013	-0.00211	4.8E-06	-0.00211	4.8E-06

RESIDUAL OUTPUT

Observation	*Predicted miR-155-5p*	*Residuals*
1	-7.63688	-0.97789
2	-7.09091	-1.1216
3	-8.68004	-1.87739
4	-7.87507	1.057317
5	-8.26503	0.322007
6	-7.38734	1.784239
7	-8.35438	-0.28931
8	-8.3636	-1.56476
9	-7.82168	1.138211
10	-7.77412	-0.31111
11	-7.63869	2.079141
12	-9.58284	-0.47534
13	-8.13041	-0.30203
14	-8.53275	-0.5736
15	-9.01342	0.007107
16	-9.04306	0.232908
17	-8.02984	0.080448
18	-8.32678	1.023093
19	-9.69003	-0.58154
20	-8.98935	0.839991
21	-7.66866	-0.49299
22	-8.61935	0.337365
23	-8.14994	-1.55271
24	-8.51903	-0.33877
25	-8.87774	0.417501
26	-9.55239	-0.61117
27	-8.34105	-0.71359
28	-8.28647	-0.37762
29	-7.85385	-0.39888
30	-8.1323	-0.41274
31	-8.16617	0.369824
32	-8.52824	-0.80452
33	-8.19083	0.935996
34	-8.18213	0.140479
35	-7.18214	-2.28706
36	-7.75617	-0.80504
37	-8.79238	0.788179
38	-7.04718	-1.32019
39	-7.88673	0.83611
40	-8.06003	1.443776
41	-7.26807	-0.45026
42	-9.04434	0.814074

43	-7.99408	-0.65408
44	-8.05027	0.612332
45	-7.46183	1.629129
46	-8.11047	1.693823
47	-8.87818	0.532198
48	-8.24383	-0.09906
49	-7.71918	-0.43545
50	-7.87204	1.532717
51	-7.966	-1.15372
52	-8.00117	0.122702
53	-8.23968	0.441262
54	-9.11567	-1.1174
55	-8.28647	-0.52587
56	-7.84297	-0.29999
57	-8.00545	1.485923
58	-7.2232	0.208862
59	-7.96716	2.269769
60	-7.65921	0.152234
61	-8.09787	-1.08203
62	-8.42882	0.268661
63	-8.37261	-1.38413
64	-8.45218	0.428933
65	-8.12305	0.817384
66	-8.426	0.13188
67	-7.24773	-1.58372

Supplementary table 3: Immune subset analysis

PID	Class7	Class4	miR-382-5p	miR-155-5p	Hb	Platelet	WBC	TLC	CD4	CD4%	CD8_impute	*Predicted miR-382-5p*	*Residuals*	CD8
2	Amb.ART	Amb	-13.68	-8.61	14.8	242	4.9	2	299	15	1239	-10.8898	-2.79495	
5	Amb.ART	Amb	-9.67	-8.21	14.8	225	7.2	3.2	327	10	1239	-11.0195	1.353612	
8	Amb.ART	Amb	-12.01	-10.56	14.7	236	8.6	1.8	417	23	1239	-10.4734	-1.54073	
9	Amb.ART	Amb	-9.55	-6.82	12.9	350	12.4	3.6	766	21	1239	-11.3843	1.833175	
34	Amb.ART	Amb	-12.95	-7.94	17.3	152	8.6	3.4	493	15	1988	-11.0136	-1.93609	1988
42	Amb.ART	Amb	-7.05	-5.60	11.4	355	5.8	1.9	195	10	1307	-10.0543	3.007823	1307
44	Amb.ART	Amb	-11.96	-8.64	10.3	388	11.9	2.5	583	23	1061	-10.1791	-1.78123	1061
3	Amb.N	Amb	-11.61	-9.93	11.5	272	6	1.6	329	21	1239	-11.0538	-0.5546	
31	Amb.N	Amb	-11.07	-6.68	13.5	256	7.1	2.1	361	17	1167	-10.2766	-0.7969	1167
45	Amb.N	Amb	-10.30	-8.09	15.4	238	7.2	2.4	281	12	1487	-9.95216	-0.34641	1487
48	Amb.N	Amb	-9.99	-5.56	9.5	190	3.3	1.6	287	18	1175	-10.8139	0.827673	1175
51	Amb.N	Amb	-10.19	-10.06	11.9	265	7.5	2.4	1164	49	628	-11.5806	1.385701	628
57	Amb.N	Amb	-12.76	-8.43	13.1	202	13.6	4.4	469	11	2000	-10.6831	-2.07604	2000
58	Amb.N	Amb	-8.92	-9.11	11.6	283	5.6	1.7	516	30	610	-10.0293	1.106242	610
64	Amb.N	Amb	-9.64	-9.01	14.7	330	11.4	2.7	909	34	968	-10.0688	0.425826	968
6	LTNP	LTNP	-13.71	-8.81	13.7	342	7	2.6	821	32	1391	-12.0065	-1.70033	
11	LTNP	LTNP	-9.59	-7.95	11.4	335	5.9	2.2	525	24	913	-11.2383	1.652194	913
12	LTNP	LTNP	-13.32	-7.30	11.7	281	6.6	2.9	822	28	1431	-12.4239	-0.89981	1431
20	LTNP	LTNP	-10.67	-10.27	15.2	397	10.2	1.5	517	34	610	-9.39931	-1.27559	610
30	LTNP	LTNP	-13.52	-8.15	12.6	233	6.4	2.4	852	36	1097	-11.7074	-1.81194	1097
36	LTNP	LTNP	-12.43	-8.16	16	178	8.7	3.4	559	16	1484	-10.8703	-1.55801	1484
38	LTNP	LTNP	-12.37	-8.28	13	284	9.3	3.5	1134	32	1732	-12.4669	0.098209	1732
62	LTNP	LTNP	-9.58	-9.70	15.2	229	6.5	2.2	448	20	1271	-9.8977	0.315056	1271
72	LTNP	LTNP	-10.87	-8.86	12.8	394	12	4.4	1124	26	2000	-11.7849	0.912146	2000
75	LTNP	LTNP	-11.15	-8.46	12.6	355	9.4	2.5	825	33	925	-10.0946	-1.05484	925
4	LTNP.NC	LTNP	-9.42	-10.16	17.9	223	4.1	1.2	345	28	1391	-10.5374	1.120504	
13	LTNP.NC	LTNP	-10.44	-9.05	12.6	291	9	2.2	550	25	955	-10.7481	0.310168	955
14	LTNP.NC	LTNP	-12.58	-8.66	16.4	318	13	3.6	830	23	1391	-11.0255	-1.55521	
23	LTNP.NC	LTNP	-12.26	-8.25	12.8	245	8.2	0	500	16	2000	-10.4708	-1.78854	2000
25	LTNP.NC	LTNP	-12.73	-8.55	11.4	143	4.8	2.2	562	26	1155	-11.6476	-1.07743	1155
37	LTNP.NC	LTNP	-12.32	-7.80	14.8	179	9.2	4.1	936	23	2000	-12.384	0.067703	2000
41	LTNP.NC	LTNP	-9.51	-9.33	12.9	349	9.1	3.4	716	21	1913	-11.544	2.036215	1913
15	Rapid	Rapid	-10.44	-7.25	10.8	424	5	0.4	65	14	1313	-9.85634	-0.58182	
16	Rapid	Rapid	-11.07	-8.04	10.5	189	4.8	1.2	225	19	1313	-10.8949	-0.17674	
17	Rapid	Rapid	-13.94	-9.47	12.6	176	4	1.9	334	17	862	-10.8756	-3.06361	862
19	Rapid	Rapid	-14.28	-8.56	11.5	256	7.2	4	475	12	2000	-12.3089	-1.97564	2000

21	Rapid	Rapid	-8.07	-8.00	11.3	350	4.8	1.3	370	29	624	-10.3421	2.271243	624
26	Rapid	Rapid	-13.04	-8.37	9.7	335	5	1.7	185	11	1001	-10.4097	-2.63438	1001
39	Rapid	Rapid	-10.74	-7.05	14.6	163	6.5	2.9	329	11	2000	-11.0213	0.283975	2000
50	Rapid	Rapid	-7.73	-6.62	13.7	267	6.7	2.3	367	16	1512	-10.2852	2.558925	1512
69	Rapid	Rapid	-9.49	-7.72	12.2	80	4.2	1.7	238	14	1198	-9.99562	0.503955	1198
1	Reg.ART	Reg	-10.12	-8.23	16	264	7.1	1.8	472	26	1329	-10.8031	0.679517	
7	Reg.ART	Reg	-8.32	-8.65	13.3	252	7.1	1.4	221	15	1329	-10.344	2.025833	
18	Reg.ART	Reg	-7.18	-7.44	11.5	134	10.2	3.2	545	16	2000	-11.9352	4.750568	2000
22	Reg.ART	Reg	-7.75	-5.83	13.7	165	6.2	2.1	339	16	1086	-10.5771	2.824429	1086
24	Reg.ART	Reg	-7.10	-6.42	13.7	167	4.5	1.1	270	25	446	-9.83245	2.732471	446
27	Reg.ART	Reg	-13.08	-8.35	10.6	248	4.9	1.2	372	31	597	-10.4033	-2.67604	597
28	Reg.ART	Reg	-11.67	-8.34	12.1	331	7.3	2.5	600	24	1171	-11.0671	-0.60242	1171
29	Reg.ART	Reg	-11.77	-8.15	11.9	211	7.3	2.4	308	13	1562	-10.8408	-0.93053	1562
32	Reg.ART	Reg	-13.46	-6.34	12.6	238	10.9	4.3	396	9	2000	-11.3432	-2.11277	2000
35	Reg.ART	Reg	-11.84	-9.12	12.5	221	4	1.4	287	21	886	-10.2365	-1.6007	886
40	Reg.ART	Reg	-8.21	-7.88	12.9	346	8.7	2.4	421	18	1126	-10.0556	1.843777	1126
47	Reg.ART	Reg	-8.37	-7.80	12.1	274	8.2	2.8	423	15	1892	-10.9143	2.549254	1892
59	Reg.ART	Reg	-10.15	-10.23	15	223	5.2	1.7	545	32	931	-9.9965	-0.15324	931
70	Reg.ART	Reg	-11.68	-8.81	14	237	7.8	3	488	16	1935	-10.5535	-1.12555	1935
71	Reg.ART	Reg	-10.75	-8.14	12.8	247	8.8	4.1	372	9	2000	-10.7356	-0.01399	2000
73	Reg.ART	Reg	-8.82	-6.52	13.6	199	6.8	1.9	343	18	1224	-9.52063	0.697767	1224
74	Reg.ART	Reg	-9.38	-7.01	9.8	229	3.1	1.1	186	17	608	-9.41344	0.029097	608
10	Reg.N	Reg	-7.47	-5.70	13	227	7.5	3	541	18	1637	-11.8046	4.333868	1637
46	Reg.N	Reg	-11.69	-7.51	13.2	177	5.4	2.1	287	14	1436	-10.4391	-1.24784	1436
56	Reg.N	Reg	-10.04	-9.18	14.7	332	6.3	2.4	521	22	1024	-10.0224	-0.02088	1024
61	Reg.N	Reg	-8.03	-8.16	15.2	194	5.7	1.4	321	23	935	-9.28738	1.257223	935
63	Reg.N	Reg	-9.47	-9.76	13.2	256	8.7	2.9	481	17	1824	-10.5187	1.051936	1824
65	Reg.N	Reg	-11.74	-8.02	12.4	155	4.8	1.7	445	26	1080	-10.2535	-1.48313	1080
66	Reg.N	Reg	-10.47	-7.31	10.1	289	8.7	2.2	544	25	803	-9.98757	-0.48477	803
67	Reg.N	Reg	-11.46	-8.29	13.4	354	6.2	2.5	422	17	1760	-10.3065	-1.15095	1760
68	Reg.N	Reg	-12.81	-8.83	0	166	6.5	3.1	377	12	1937	-12.5524	-0.26243	1937
